# Glial remodeling enhances short-term memory performance in Wistar rats

**DOI:** 10.1186/s12974-020-1729-4

**Published:** 2020-02-07

**Authors:** Simone N. De Luca, Alita Soch, Luba Sominsky, Thai-Xinh Nguyen, Abdulhameed Bosakhar, Sarah J. Spencer

**Affiliations:** 1 School of Health and Biomedical Sciences RMIT University, Melbourne, VIC 3083 Australia; 2 ARC Centre of Excellence for Nanoscale Biophotonics, RMIT University, Melbourne, VIC Australia

**Keywords:** Astrocytes, Cognition, Golgi, Microglia, Transgenic rat

## Abstract

**Background:**

Microglia play a key role in neuronal circuit and synaptic maturation in the developing brain. In the healthy adult, however, their role is less clear: microglial hyperactivation in adults can be detrimental to memory due to excessive synaptic pruning, yet learning and memory can also be impaired in the absence of these cells. In this study, we therefore aimed to determine how microglia contribute to short-term memory in healthy adults.

**Methods:**

To this end, we developed a *Cx3cr1*-*Dtr* transgenic Wistar rat with a diphtheria toxin receptor (*Dtr*) gene inserted into the fractalkine receptor (*Cx3cr1*) promoter, expressed on microglia and monocytes. This model allows acute microglial and monocyte ablation upon application of diphtheria toxin, enabling us to directly assess microglia’s role in memory.

**Results:**

Here, we show that short-term memory in the novel object and place recognition tasks is entirely unaffected by acute microglial ablation. However, when microglia repopulate the brain after depletion, learning and memory performance in these tasks is improved. This transitory memory enhancement is associated with an ameboid morphology in the newly repopulated microglial cells and increased astrocyte density that are linked with a higher density of mature hippocampal synaptic spines and differences in pre- and post-synaptic markers.

**Conclusions:**

These data indicate that glia play a complex role in the healthy adult animal in supporting appropriate learning and memory and that subtle changes to the function of these cells may strategically enhance memory.

## Background

Normal executive function, including learning and memory, is mediated by acute and long-term dynamic regulation of neuronal connectivity. In particular, the hippocampus is actively involved in memory acquisition, formation, and maintenance [1–6], with adult neurogenesis and dendritic spine density in this region being particularly important in these functions [7, 8]. However, the focus on mechanisms coordinating cognition has, until recently, been almost exclusively on neurons. Evidence now suggests that microglia may be an important, if under-appreciated, player in these processes.

Microglial cells are a major immune cell population in the brain that coordinate synaptic pruning during development and central responses to pathogens and brain injury in the mature animal [9–13]. It is clear that appropriate synaptic pruning and clearance of excess neurons by microglia during early development is important for the maturation of neural circuits, for instance, with microglial knockout mice displaying immature excitatory synapse functioning [14–16]. On the other hand, the role of microglia in learning and memory in the mature animal is less clear.

In the normal adult brain, microglia play a key role in neurogenesis by phagocytosing apoptotic cells [17], controlling the increased neurogenesis caused by environmental enrichment [18], and decreasing neurogenesis following inflammatory challenges [19]. However, a recent surprising finding is that mice perform better on the Barnes maze task for spatial learning and memory in the near complete absence of these cells [20]. In our own work, we have shown that rats that do not effectively reduce microglial number and density in response to a learning task (the radial arm maze) have poorer performance in that task than controls [21]. Learning and memory can also be impaired in mild, and, of course, major neuroinflammation when these cells are at their most active [8, 22]. Conversely, short-term microglial depletion in adult CX_3_CR1^CreER^ mice can impair motor learning in a rotarod task, stimulate a reduction in synapse formation in motor learning-induced remodeling, and reduce recognition memory in a novel object recognition (NOR) task [23]. Thus, while microglia are necessary for many important functions in the mature animal, including effective responses to pathogens and injury [15, 24], they may play a more nuanced modulatory role in cognitive performance and may curtail or contribute to effective learning under specific conditions.

In this study, we hypothesized that acute and prolonged conditional microglial ablation in a rat model would lead to improvements in learning and memory and that this would be associated with changes in synaptic pruning and neuronal maturation. We used a transgenic rat with the diphtheria toxin receptor (*Dtr*) inserted into the promoter region of the fractalkine receptor, *Cx3cr1*, expressed on microglia and monocytes, to allow temporary ablation of microglia upon application of diphtheria toxin (DT). We have previously established that microglial ablation in this model does not induce sickness or an increase in peripheral or central cytokines [22]. We examined performance in several behavioral tasks of learning and memory, and their neurobiological correlates in the hippocampus after microglial ablation and repopulation. We demonstrate that microglial ablation does not affect memory, but memory is improved as these cells repopulate; an effect that may be linked to changes in astrocyte density.

## Methods

### Animals

We conducted all experiments in accordance with the Australian Code of Practice for the Care and Use of Animals for Scientific Purposes, with approval from the RMIT University Animal Ethics Committee.

For these experiments, we utilized a knock-in rat model, on a Wistar Han background, in which a diphtheria toxin receptor (*Dtr*) sequence is expressed under the control of the endogenous *Cx3cr1* promoter sequence. The fractalkine receptor, *Cx3cr1*, is expressed on microglia and monocytes. These rats, originally produced for us by SageLabs Inc., a subsidiary of Horizon Discovery Limited (St Louis, MO, USA), have been described previously [22]. We have previously demonstrated that the *Dtr* insertion is inert in the absence of diphtheria toxin (DT), not affecting any measured parameters including litter size, neonatal or adult weights, circulating and spleen monocytes, hypothalamic *Cx3cr1* expression, hypothalamic microglia, or exploratory behaviors [22].

**Fig. 1:**
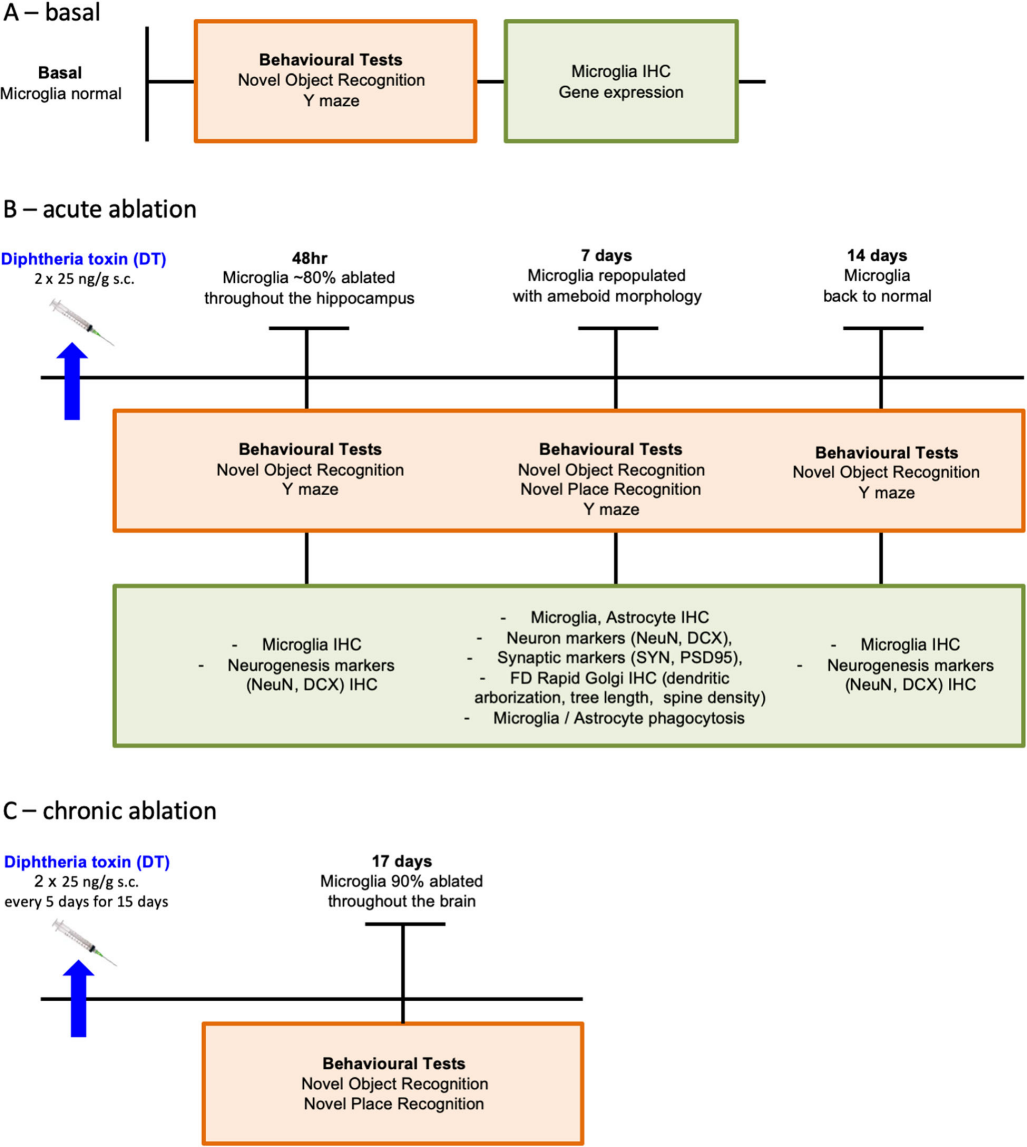
**a** Basal conditions experiment. **b** Acute ablation experiment. **c** Chronic ablation experiment

In these experiments, we used 146 male rats aged between 9 and 12 weeks. We chose males since our original data illustrating microglial changes in association with cognitive deficits in the neonatally overfed were in males [21]. Our previously published analyses suggest female *Cx3cr1*-*Dtr* rats perform similarly in terms of microglial and weight responses to the DT [22] and effects on memory in females will be the subject of future investigation. Specific sample sizes are indicated in the figure legends. The rats were kept under standard laboratory housing conditions, with a 12 h light cycle (7 am to 7 pm), an ambient temperature of 22 °C, with humidity between 40 and 60%, and free access to water and standard rat chow. To acutely ablate microglia, we gave DT (from *Corynebacterium diphtheria*, Sigma-Aldrich, St Louis, MO, USA) to wildtype (wt) and *Cx3cr1*-*Dtr* rats as two subcutaneous (s.c.) injections, 8 h apart, of 25 ng/g DT in sterile saline. Rats were allowed to recover for 48 h, 7 days, or 14 days before behavioral testing and cull. To chronically ablate microglia, DT was administered every 5 days for 15 days (25 ng/g DT in sterile saline, s.c.). In this case, rats were allowed to recover for 2 days before behavioral testing and cull (Fig. 1). We collected post-mortem tissue, after the rats had been deeply anesthetized with 150 mg/kg intraperitoneal (i.p.) sodium pentobarbital.

### Behavior

#### Y maze

To assess if microglial depletion could influence spatial memory, we tested the rats in a Y-maze task, which exploits the rats’ natural tendency to explore novel environments [25], as we have described previously [21]. The testing protocol involved a 10-min exposure, 1-h inter-trial interval (ITI), and a 5-min test phase. An experimenter blinded to treatment groups assessed the number of entries into and the time spent in each arm. Data are expressed as a discrimination index calculated as the number of entries into the novel arm minus the number of entries into the non-entry familiar “open” arm divided by the total arm entries (i.e., the analysis excludes the “home” arm the rat is initially placed into).

#### NOR

To examine possible effects on recognition memory, we next tested a separate cohort of rats in the NOR task [26] as we have previously described [21]. This test also exploits the rats’ attraction to novelty, in this case a novel object in the same context. The testing protocol involved a 3-min acquisition trial, a 1-h ITI, and a 3-min retention trial. Results from the retention trial are expressed as a discrimination index calculated as the time spent interacting with the novel object minus the time spent interacting with the familiar object divided by the overall exploration time of the two objects in seconds.

#### Novel place recognition

To further assess the rats’ spatial memory, we exposed them to a novel place recognition (NPR) task. The task was similar to the NOR except that during the retention phase, rather than replacing a familiar object with a novel object, one of the (identical) objects was moved to a novel position.

### Microglia, astrocyte, and neuronal assessment

To determine microglial, astrocyte, and neuron numbers and morphology under basal conditions and at the various time points after DT, we collected whole brains and immersion-fixed them in 4% paraformaldehyde (PFA) for 24 h before cryoprotecting in 20% sucrose for at least a further 24 h. We then cut the brains into 30 μm coronal sections using a cryostat and subsequently processed the sections using immunohistochemistry for ionized calcium binding adaptor molecule 1 (Iba-1) and other markers. We confined our analyses principally to the hippocampus because of its involvement in recognition and spatial memory [23], but also assessed microglial changes in five other regions important for cognition.

For the immunohistochemistry, we incubated a one in five series of 30 μm sections (150 μm apart) from each animal in primary antibody overnight (Iba-1: 1:1000, rabbit, 4 °C, Wako Chemicals, USA Inc., Richmond, VA, USA. Neuronal nuclei (NeuN): 1:5000, rabbit, 4 °C, Abcam, Cambridge, England, UK. Doublecortin (DCX): 1:500, goat, 4 °C, Santa Cruz Biotechnology Inc., Dallas, TA, USA. Glial fibrillary acidic protein (GFAP): 1:1000, rabbit, 4 °C, Dako, Glostrup, Denmark. Synaptophysin: 1:1000, mouse, room temperature (RT), Sigma-Aldrich. Post-synaptic density (PSD)-95: 1:200, rabbit, 4 °C, Life Technologies, Carlsbad, CA, USA). This was followed by secondary antibody (Iba-1: 1.5 h, 1:200, biotinylated anti-rabbit, Vector Laboratories, Burlingame, CA, USA. DCX: 1.5 h, 1:500, biotinylated anti-goat, Vector. GFAP: 2 h, 1:500, Alexa-Fluor-594 anti-mouse, Life Technologies. Synaptophysin: 2 h, 1:500, Alexa-Fluor-594 anti-mouse, Life Technologies. NeuN, PSD-95: 2 h, 1:500, Alexa-Fluor-488 anti-rabbit, Life Technologies). For Iba-1 and DCX, we used an avidin-biotin horseradish peroxidase (HRP) complex (ABC; 45 min; Vector Elite kit; Vector) followed by diaminobenzidine to visualize the HRP activity, seen as amber staining. We mounted the sections on gelatin-coated slides, air-dried them, dehydrated them in a series of alcohols, cleared them in histolene, and coverslipped. For NeuN, GFAP, synaptophysin, and PSD-95, we mounted the sections on SuperFrost slides and coverslipped them with Fluoroshield with Dapi mounting medium (Sigma-Aldrich). Sections were processed in batches with representative animals from each experimental group in each batch.

We assessed sections through the brain in a blinded fashion for numbers and morphology of cells positive for Iba-1. For each region, we selected a sub-region of interest (ROI), identified according to the Paxinos and Watson Rat Brain Atlas [27], and analyzed four to five sections as our sampled results for each animal (hippocampus: four sections between 2.52 and 4.36 mm caudal to the bregma; retrosplenial cortex (RsC): four sections between 2.64 and 4.56 mm caudal to the bregma; perirhinal cortex: five sections between 3.00 and 4.80 mm caudal to the bregma; basolateral (BLA) and central amygdala (CeA): four sections between 2.04 and 3.36 mm caudal to the bregma). We took the summed counts of these sections as our sampled result for each animal. For morphology in the 7- and 14-day groups, all cells were categorized as ameboid (0–1 primary process), intermediate (2–4 primary processes), or ramified (five or more primary processes). We also assessed microglial complexity with a Sholl analysis on three cells per section from the same images as described in detail in [28]. Data were grouped into bins of 1–10 or 1–20 μm for analysis with repeated measures analyses of variance (ANOVA) followed, where significant, by Student’s unpaired *t* tests.

For numbers of mature neurons, we manually counted NeuN-positive cells in the CA1 and CA3 ROIs and conducted thresholding with the Image J software (National Institutes of Health (NIH), Bethesda, MD, USA) for the dentate gyrus. For immature neurons, we manually counted DCX-positive cells in the subgranular/granular region of the dentate gyrus. No DCX-immunoreactive cells were visible elsewhere. For GFAP, synaptophysin, and PSD-95, we employed the thresholding method on ROIs through the hilus (synaptophysin, PSD-95) or all the subregions of the hippocampus (GFAP) to determine the intensity of immunofluorescence measured as an integrated density using ImageJ.

### Gene expression

To assess changes in gene expression, we dissected hippocampi over ice from wt and *Cx3cr1*-*Dtr* rats. The samples were immediately snap-frozen in liquid nitrogen and stored at − 80 °C until use in quantitative rt-PCR (qrt-PCR) as we have previously described [21, 29–31]. We compared a relative quantitative measure of the target gene expression with an endogenous control, *Gapdh*. See Table 1 for primer details. We analyzed mRNA expression using the equation 2^-ΔΔ*C*(*t*)^, where *C*(*t*) is the threshold cycle at which fluorescence is first detected significantly above background [32]. Data are presented as a fold increase relative to wt.

**Table 1:** TaqMan probe details (Life Technologies) used for qrt-PCR

Target gene	NCBI reference sequence	TaqMan assay ID	Product size
*Bdnf*	NM_001270630.1	Rn0251967_s1	142
*C1qa*	NM_001008515.1	Rn01519903_m1	117
*C3*	NM_016994.2	Rn00566466_m1	72
*Cd14*	NM_021744.1	Rn00572656_g1	56
*Crybb1*	NM_012936.2	Rn00564028_m1	81
*Csf1*	NM_023981.4	Rn01522726_m1	115
*Cx3cl1*	NM_134455.1	Rn00593186_m1	74
*Cx3cr1*	NM_133534.1	Rn02134446_s1	124
*Cxcl10*	NM_139089.1	Rn01413889_g1	106
*Cxcr2*	NM_017183.1	Rn02130551_s1	122
*Dcx*	NM_053379.3	Rn00670390_m1	72
*Gapdh*	NM_012512.2	Rn00560865_m1	58
*Gfap*	NM_017009.2	Rn01253033_m1	75
*Gria2* (*Glua2*)	NM_001083811.1	Rn00568514_m1	122
*Grin2a* (*Glun2a*)	NM_012573.3	Rn00561341_m1	68
*Lyz2*	NM_021771.3	Rn00562794_m1	92
*Mafb*	NM_019316.1	Rn00709456_s1	70
*Mcm5*	NM_001106170.1	Rn01536832_m1	59
*Mef2a*	NM_001014035.1	Rn01478096_m1	118
*Nkt2* (*Ngf*)	NM_001163168.2	Rn01441749_m1	73
*Rbfox3* (*Neun*)	NM_001134498.2	Rn01464214_m1	82
*Vglut1*	NM_053859.2	Rn01462431_m1	71

### Golgi staining and analysis

To assess changes in neuronal branching and dendritic spines, we dissected 10-mm-thick brain slices that included hippocampi from a separate cohort of wt and *Cx3cr1*-*Dtr* rats 7 days after acute microglial ablation. Dissections were completed over ice and we then conducted Golgi Cox staining using the FD Rapid GolgiStain kit (FD Neurotechnologies, Columbia, MD, USA) according to the manufacturer’s instructions. In preparation for sectioning, the tissue was rapidly frozen in isopentane pre-cooled on dry ice. Coronal sections of 100 μm thickness were cut and transferred to gelatin-coated slides. After allowing the sections to dry at RT in the dark overnight, slides were then stained with FD Solution and coverslipped with DPX (Sigma-Aldrich).

Dendritic branching and spine densities of pyramidal neurons in the hippocampus (subfields CA1 and CA3) were assessed by an experimenter blinded to treatment groups. We measured pyramidal neuron basal and apical dendritic tree lengths as well as basal dendritic tree arborization. To analyze basal dendritic spines, photomicrographs were taken on an upright microscope (Olympus BX61, Notting Hill, VIC, AUS) at × 100 magnification with a step-width of 0.7 μm using the z-stack option of the imaging software (CellSens, Olympus). For each animal, four sections were used to generate eight z-stacks of secondary dendritic segments (minimum of 15 μm), allowing us to analyze 18 to 28 dendritic segments per animal. To ensure that a uniform population of dendrites and spines was analyzed within and across subjects, we ensured that (1) cell bodies were within the area of interest, (2) the length of the branch was unbroken, and (3) the length of the dendrite was isolated well enough for an unobstructed view. We only analyzed segments with spines that were fully attached to the dendrite, avoiding segments with spines whose structure was not completely visible. Using Imaris software (Bitplane, Concord, MA, USA), spines were measured and characterized according to their morphology. For spines to be included in our analyses, a maximum spine length and minimum spine end diameter was set at 2.5 μm and 0.3 μm, respectively. Protrusions were distinguished into six categories and expressed as the mean number of spines per 10 μm for each rat: (1) filopodia with no detectable head (> 2 μm); (2) long thin spines with a long neck (< 2 μm); (3) thin spines (< 1 μm) with a long neck (< 0.6 μm); (4) mushroom spines with a short neck (> 0.6 μm); (5) stubby spines with a head but without a neck (< 0.5 μm); and (6) bifurcated spines with two heads [33]. Data are expressed both per dendritic segment and per animal [34].

### Microglial and astrocyte phagocytosis

To assess changes in microglial and astrocyte phagocytosis, we anesthetized wt and *Cx3cr1*-*Dtr* rats 7 days after administering DT as described above with 20 mg/mL ketamine, 5 mg/mL xylazine. Upon deep anesthesia, an incision was made in the abdomen to expose the thoracic cavity allowing us to perform a cardiac perfusion with ice-cold sucrose substituted artificial cerebrospinal fluid (saCSF containing in mM: 250 sucrose, 25 NaHCO_3_, 11 glucose, 2.5 KCl, 1 NaH_2_PO_4_, 6 MgCl_2_, and 1 CaCl_2_; pH 7.3) to eliminate circulating macrophages and monocytes. Rats were then decapitated, brains removed, and blocked coronally between 2.92 and 3.52 mm caudal to the bregma. We took 4 × 150 μm sections through the hippocampus using a vibrating microtome (VT1200s, Leica Microsystems, Nuslock, Germany). Slices were transferred to a nylon grid chamber with oxygenated aCSF (aCSF containing in mM: 118 NaCl, 25 NaHCO_3_, 11 glucose, 2.5 KCl, 1 NaH_2_PO_4_, 1 MgCl_2_, and 2.5 CaCl_2_) and allowed to equilibrate for 1 h at room temperature (22–25 °C). During this time, yellow-green fluorescently labeled microspheres (Fluoresbrite, 2 μm diameter, Polysciences Europe GmbH, Eppelheim, Germany) were diluted 1:100 in fetal calf serum (FCS, Sigma-Aldrich) to achieve a 1% suspension. The suspension was agitated on a plate shaker for 30 min then centrifuged (2 min at 3000 rpm at 4 °C) to pellet the beads. The solution was aspirated and replaced with the oxygenated aCSF and the beads resuspended. Following completion of incubation, brain slices were transferred into a 12-well plate, where they were incubated with 1 mL of the bead suspension for 2 h at 37 °C. They were then washed 3 × 5 min with ice cold 1× PBS, fixed in 4% paraformaldehyde for 1 h, washed again 3 × 5 min with ice cold 1× PBS, and then mounted onto SuperFrost slides. Sections were dried onto the slides overnight at 37 °C before processing for immunofluorescence as described above. We took photomicrographs from regions of interest within the hippocampus on a multiphoton confocal microscope (A1-MP Nikon) using a × 20 objective and NIS-Element Viewer software (Nikon). We initially used Imaris imaging software (Bitplane) to create reconstructions of the cells to verify true co-localization of the beads within the cell. Bead internalization was verified for each image by imaging the region as a z-stack, transforming the z-stack into a 3D image, and visually ensuring that the maximum fluorescence intensity started from within the cell. We assessed numbers of Iba-1 or GFAP-positive cells containing fluorescently labeled microbeads in four sections per animal throughout the region of interest. The summed counts of microglia or astrocytes and cells colocalized with beads were used as our sampled result.

### Data analysis

We assessed our data for statistical outliers using Grubb’s test. We then assessed the data for normality of distribution using the Shapiro-Wilk’s test complemented by assessment of skewness and kurtosis. Since the microglial morphology data (Figs. 4 and 6) commonly violated normality of distribution, we analyzed these data using Mann-Whitney *U* tests. All other data were also assessed for homogeneity of variance using Levene’s test and were analyzed using Student’s unpaired *t* tests or two-way ANOVAs with genotype and day (recovery time) as between factors, with a correction for unequal distribution where appropriate. Where significant interactions were found, we then performed Tukey post hoc tests. Data are presented as the mean ± SEM. Statistical significance was assumed when *p* ≤ 0.05.

## Results

### The Cx3cr1-Dtr rat allows selective inducible, reversible ablation of microglia

As we have previously reported for other behaviors and brain regions [22], hippocampal microglial numbers and hippocampal *Cx3cr1* mRNA expression were not affected under basal conditions, i.e., by the presence of the *Dtr* alone (Additional file 2: Figure S1). Memory-related behaviors in the Y maze and NOR tasks were also not affected, with both groups successfully preferring the novel arm in the Y maze test and the novel object in the NOR test (Additional file 2: Figure S1).

**Fig. 2:**
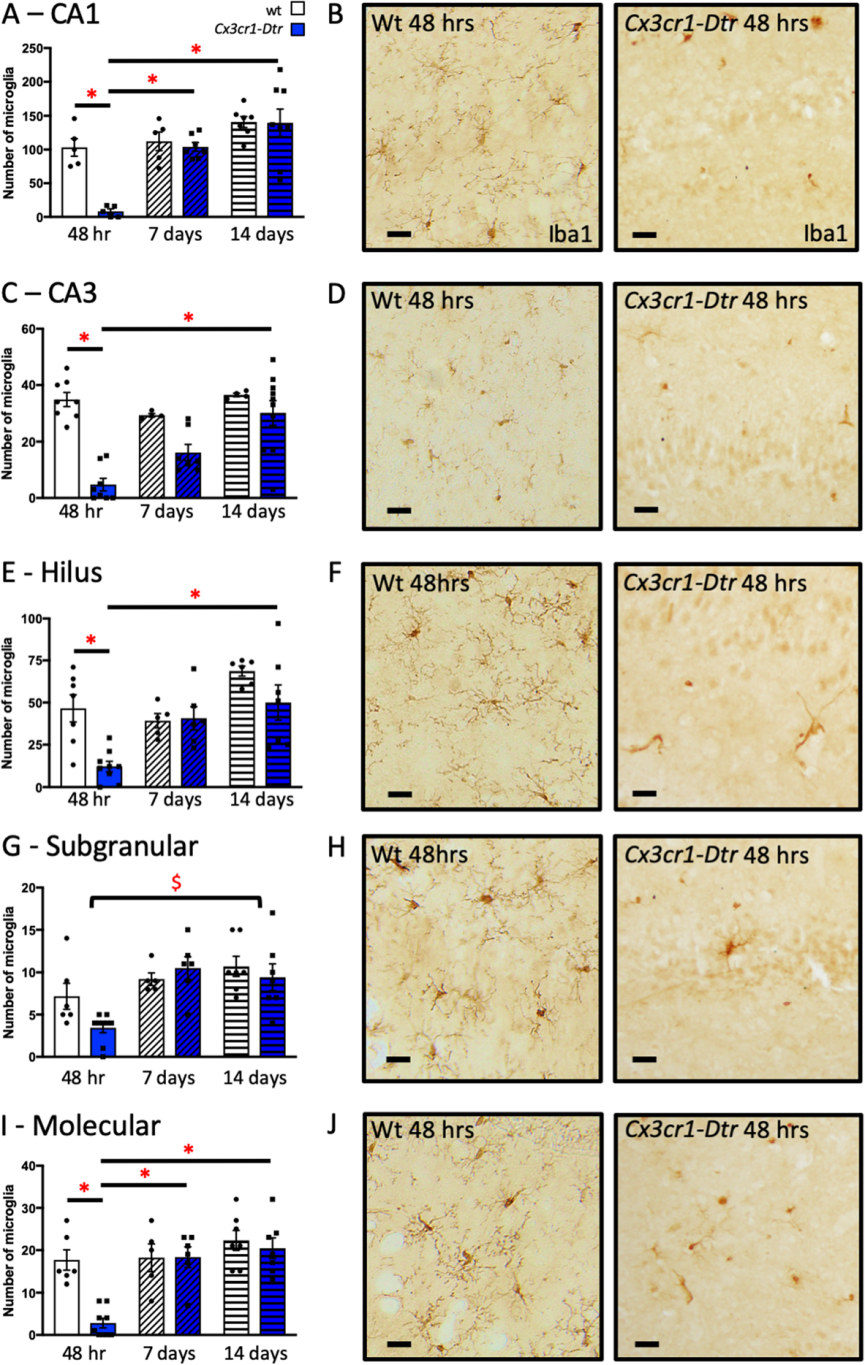
The *Cx3cr1*-*Dtr* rat allows selective inducible ablation of microglia. Numbers of microglia were reduced throughout the hippocampus by 48 h after diphtheria toxin in *Cx3cr1*-*Dtr* rats compared to wild-types (wt) with microglia repopulating the hippocampus by 7 days (*n* = 5–8 per group). **a**, **b** CA1 (significant genotype by day interaction: *F*_(2, 31)_ = 6.61, *p* = 0.004); **c**, **d** CA3 (interaction: *F*_(2, 35)_ = 6.66, *p* = 0.004); **e**, **f** hilus (interaction: *F*_(2, 34)_ = 3.53, *p* = 0.040); **g**, **h** subgranular/granular (significant effect of day: *F*_(1, 35)_ = 10.43, *p* < 0.001); or **i**, **j** molecular (interaction: *F*_(2, 34)_ = 6.61, *p* = 0.004) regions of the dentate gyrus. *p < 0.05 with Tukey post hoc tests. $ significant main effect of day (recovery time). Data are expressed as mean ± SEM. Scale bars = 10 μm

DT ablated microglia throughout the hippocampus by 48 h in our *Cx3cr1*-*Dtr* rats (Fig. 2). Iba1-positive cells were dramatically (92.5%) ablated in the CA1 and reduced by 82.8% in the CA3, with other regions assessed, including the hilus, subgranular/granular, and molecular regions of the dentate gyrus, showing effects within this range. In all regions, microglia had repopulated the brain so that numbers were not significantly different from wt by 7 days after DT.

### Microglial ablation does not acutely affect learning and memory

**Fig. 3:**
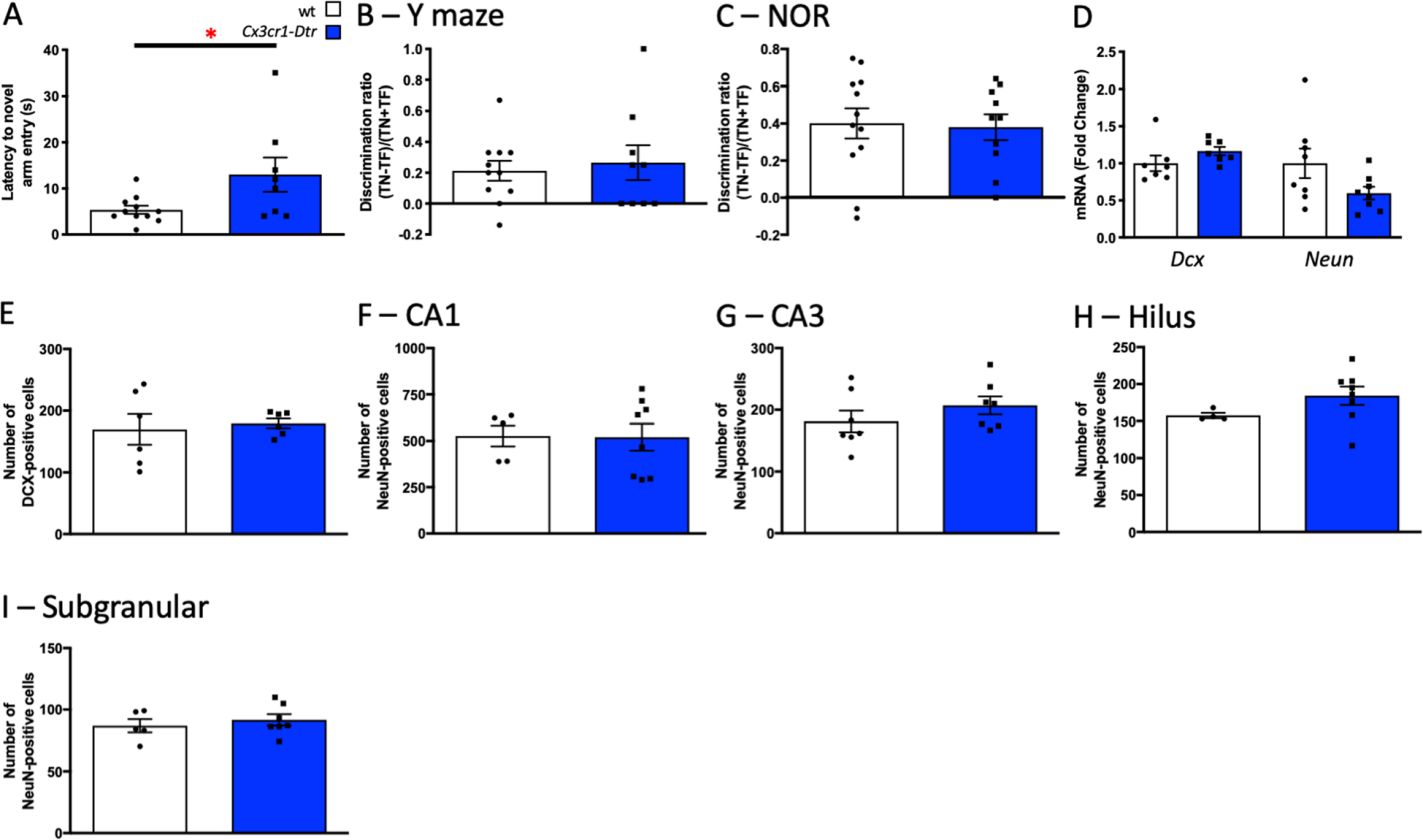
Microglial ablation does not acutely affect learning and memory or neuron numbers or survival. Memory task performance at 48 h after diphtheria toxin injection to ablate microglia in *Cx3cr1*-*Dtr* rats compared to wild-types (wt). **a** Y-maze latency to novel arm entry (*t*_(17)_ = 2.32, *p =* 0.003; *n* = 8–11). **b** Y-maze discrimination ratio (one sample *t* test to zero: wt: *t*_(10)_ = 3.31, *p* = 0.0079. *Cx3cr1*-*Dtr*: *t*_(8)_ = 2.34, *p* = 0.046). **c** Novel object recognition discrimination ratio (one sample *t* test to zero: wt: *t*_(11)_ = 4.94, *p* = 0.0004. *Cx3cr1*-*Dtr*: *t*_(9)_ = 5.43, *p* = 0.0004; *n* = 10–12). **d** Hippocampal doublecortin (*Dcx*), neuronal nuclei (*Neun*) gene expression (*n* = 6 per group). **e** Number of DCX-positive cells in the subgranular/granular region of the dentate gyrus (*n* = 6 per group). **f** Number of NeuN-positive cells in the CA1; **g** CA3; **h** hilus; **i** subgranular/granular region of the dentate gyrus. *p < 0.05: Student’s unpaired *t* tests. Data are expressed as mean ± SEM

To test the role of microglia in learning and memory, we depleted microglia with s.c. DT in our *Cx3cr1*-*Dtr* rats and assessed their performance, relative to wt controls, in two memory tests (Fig. 3). For the Y maze, at 48 h after DT (when the microglia are ablated throughout the hippocampus), the *Cx3cr1*-*Dtr* rats showed an increased latency to enter the novel arm compared with the wt controls (Fig. 3a). However, total exploration was not affected (Additional file 3: Figure S2) and both groups showed the expected preference for the novel arm, with no group differences (Fig. 3b). To assess recognition memory, we examined the rats’ performance in the NOR task. There were no differences between the *Cx3cr1*-*Dtr* and wt rats in the acquisition phase of the NOR task with rats spending equal time with both objects (Additional file 3: Figure S2). Microglial ablation also did not affect object preference in the retention phase; both groups showing similar positive discrimination ratios (Fig. 3c), together suggesting that acute microglial ablation does not affect learning and memory.

### Microglial ablation does not acutely affect neuron numbers or survival

We next investigated if acute microglial ablation was associated with changes in neuron numbers and survival by assessing numbers of cells positive for DCX, a marker of immature neurons, and NeuN, a marker of mature neurons. We found no differences in expression of *Dcx* or *Neun* mRNA in the hippocampus of microglia-depleted rats at 48 h after DT administration (Fig. 3d). There were also no differences in the number of DCX-positive cells in the subgranular/granular region of the hippocampus between wt and *Cx3cr1*-*Dtr* DT-treated animals, and no differences in NeuN-positive cells in any regions of the hippocampus (Fig. 3e–i).

### Microglial ablation improves short-term memory as the microglia repopulate the brain

**Fig. 4:**
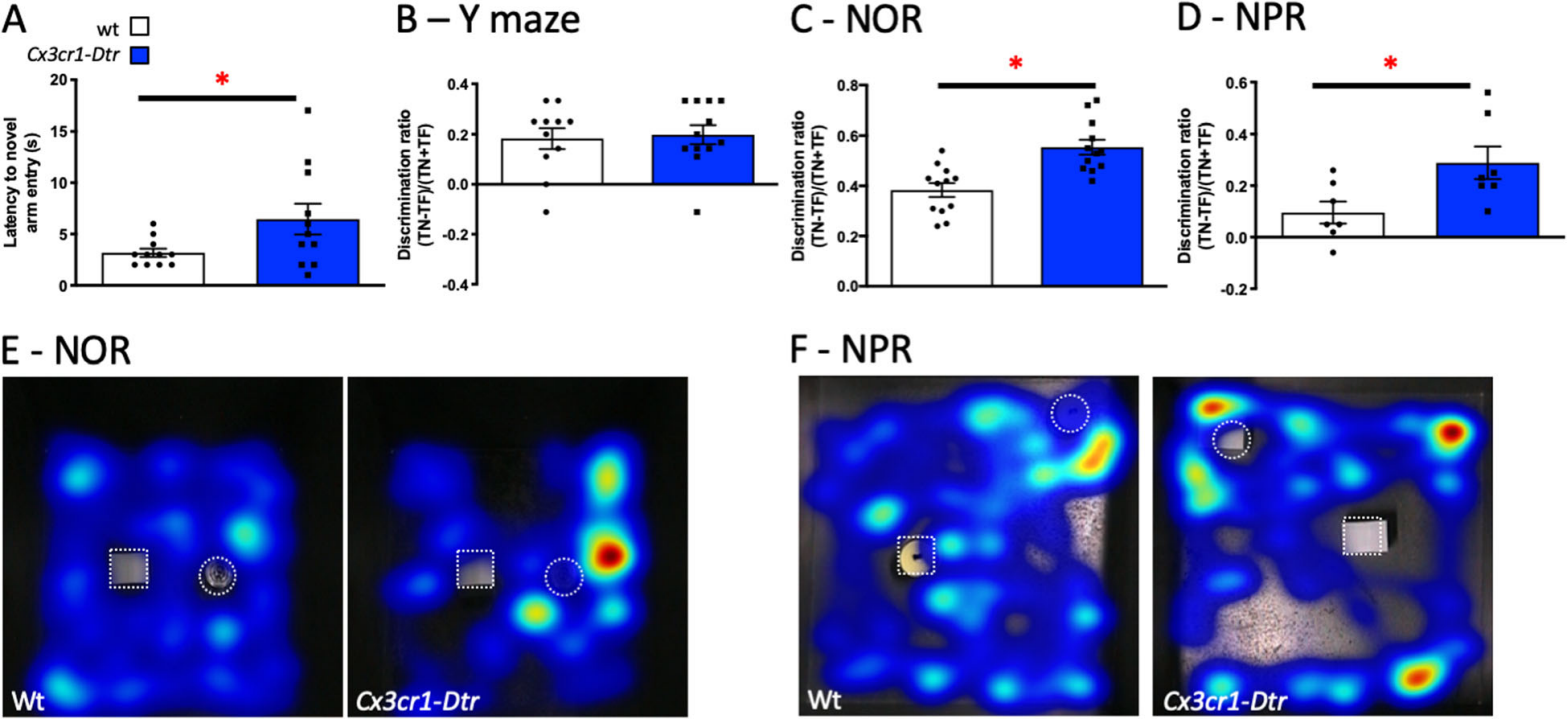
Microglial ablation improves short-term memory function as the microglia repopulate the brain. Memory task performance at 7 days after diphtheria toxin injection to ablate microglia in *Cx3cr1*-*Dtr* rats compared to wild-types (wt). **a** Y-maze latency to novel arm entry (*t*_(20)_ = 2.11, *p =* 0.048; *n* = 8–11). **b** Y-maze discrimination ratio (one sample *t* test to zero: wt: *t*_(10)_ = 4.39, *p* = 0.0013. *Cx3cr1*-*Dtr*: *t*_(11)_ = 5.24, *p* = 0.0003). **c** Novel object recognition discrimination ratio (one sample *t* tests to zero: wt: *t*_(11)_ = 13.73, *p* < 0.0001. *Cx3cr1*-*Dtr*: *t*_(11)_ = 18.72, *p* < 0.0001; *n* = 10–12. *Cx3cr1*-*Dtr* versus wt: *t*_(22)_ = 4.20, *p <* 0.001). **d** Novel place recognition discrimination ratio (one sample *t* test to zero: wt: *t*_(6)_ = 2.25, *p* = 0.066. *Cx3cr1*-*Dtr*: *t*_(6)_ = 4.59, *p* = 0.004; *n* = 7 per group. *Cx3cr1*-*Dtr* versus wt: *t*_(12)_ = 2.54, *p =* 0.026). **e** Heat maps illustrating familiar object (square) and novel object (circle) exploration in the NOR. **f** Heat maps illustrating novel place (circle) exploration in the NPR. Note, in this test both objects were identical; the superimposed circle is to show the novel position. Heat maps were generated in Ethovision using the over-heatmap setting allowing comparison between the representative animals. *p < 0.05: Student’s unpaired *t* tests. Data are expressed as mean ± SEM

To determine whether short-term learning and memory could be influenced by microglial repopulation of the brain after depletion, we tested the rats’ memory performance at 7 days after administering the DT to ablate the microglia. At this time, the *Cx3cr1*-*Dtr* rats showed an increased latency to enter the novel arm compared with the wt controls in the Y-maze (Fig. 4a). However, their overall performance in terms of total exploration (Additional file 3: Figure S2), and discrimination ratio (Fig. 4b) were similar between groups, with the expected novel arm preference.

Notably, however, microglial ablation did affect performance in the NOR test. Here, there were no differences between the *Cx3cr1*-*Dtr* and wt rats in the acquisition phase, with rats spending equal time with both objects (Additional file 3: Figure S2). Both groups of rats had a discrimination ratio that was significantly different from zero. However, *Cx3cr1*-*Dtr* rats had an increased positive discrimination ratio in the retention trial compared to wt rats, indicative of a higher preference for the novel object and thus better memory of the familiar one (Fig. 4c, e).

As the *Cx3cr1*-*Dtr* rats showed improved memory in the NOR task, we also assessed spatial memory in the NPR task. As with the NOR, there were no differences in the acquisition phase of the NPR task between the *Cx3cr1*-*Dtr* and wt rats, with both groups spending equal time with both objects (Additional file 3: Figure S2). The *Cx3cr1*-*Dtr* rats had a discrimination ratio that was significantly different from zero and the *p* value for the wt was 0.066. Again, the *Cx3cr1*-*Dtr* rats had an increased positive discrimination ratio compared to wt rats in the retention trial (Fig. 4d, f), suggesting that microglial repopulation after ablation also leads to better recall in this task relative to wt controls.

### Microglial ablation alters neuron numbers as the microglia repopulate the brain

**Fig. 5:**
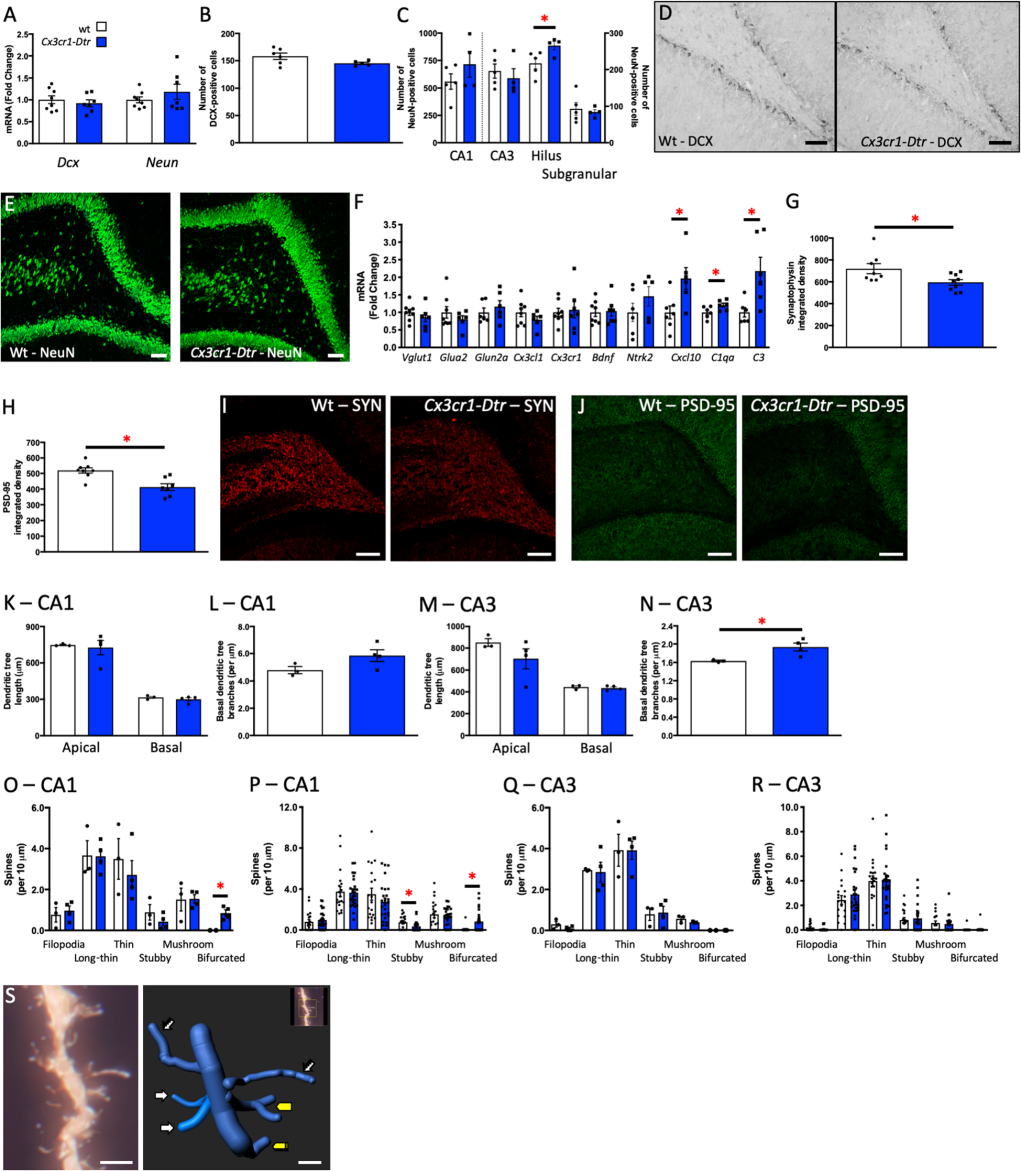
Microglial ablation alters neuronal markers as microglia repopulate the brain. Neuronal markers at 7 days after diphtheria toxin injection to ablate microglia in *Cx3cr1*-*Dtr* rats compared to wild-types (wt). **a** Hippocampal doublecortin (*Dcx*)*,* neuronal nuclei (*Neun*) gene expression. **b** Number of DCX-positive cells in the subgranular/granular region of the dentate gyrus. **c** Number of NeuN-positive cells in the CA1, CA3, hilus, subgranular/granular region of the dentate gyrus (hilus: *t*_(7)_ = 2.44, *p =* 0.044; *n* = 4–5). **d** Representative photomicrographs of DCX. **e** Representative photomicrographs of NeuN. **f** Hippocampal gene expression: *Vglut1*, *Glua2*, *Glun2a*, *Cx3cl1*, *Cx3cr1*, *Bdnf*, *Nkt2*, *Cxcl10*, *C1qa*, *C3* (*Cxcl10*: *t*_(11)_ = 2.80, *p* = 0.017; *C1q: t*_(10)_ = 2.29, *p* = 0.048; *C3: t*_(10)_ = 2.83, *p* = 0.018). **g** Synaptophysin density in the hilus (*t*_(15)_ = 2.43, *p* = 0.028). **h** Post-synaptic density (PSD)-95 density in the hilus region of the hippocampus (*t*_(13)_ = 3.87, *p* = 0.002). **i** Representative photomicrographs of synaptophysin. **j** Representative photomicrographs of PSD-95. **k** Apical and basal dendritic tree length in the CA1. **l** Basal dendritic tree branches in CA3. **m** Apical and basal dendritic tree length in the CA3. **n** Basal dendritic tree branches in CA1 (*t*_(5)_ = 2.89, *p* = 0.034; *n* = 3–4). **o** Dendritic spine density in CA1, analysis per animal (bifurcated: *t*_(4)_ = 3.60, *p* = 0.023; *n* = 2–4). **p** Dendritic spine density in CA1, analysis per dendrite (stubby: *t*_(41)_ = 2.25, *p* = 0.03; bifurcated: *t*_(41)_ = 2.95, *p* = 0.005). **q** Dendritic spine density in CA3, analysis per animal. **r** Dendritic spine density in CA3, analysis per dendrite. **s** Representative photomicrograph of Golgi-stained spines. White arrows = long-thin spines; hatched white arrows = filopodia; yellow block connector = bifurcated; yellow crossed block connector = stubby. *p < 0.05: Student’s unpaired *t* tests. Data are expressed as mean ± SEM. **d**, **e** Scale bars = 50 μm. **i**, **j** Scale bars = 100 μm. **q** Left panel scale = 2 μm, right panel scale = 1 μm

We had previously shown that spatial learning in the radial arm maze causes a reduction in neurogenesis, potentially allowing more robust memory consolidation [21]. Therefore, we hypothesized that this improved NOR and NPR memory after microglial repopulation would be associated with changes in the number of neurons in the hippocampus. There were no differences in *Dcx* or *Neun* mRNA expression in the hippocampus of microglial-depleted animals at 7 days after DT administration (Fig. 5a). Nor were there differences in the number of immature neurons in the subgranular/granular region of the dentate gyrus (Fig. 5b, d). However, microglial repopulation was associated with an increase in the number of mature neurons in the hilus (Fig. 5c, e).

### Microglial repopulation, short-term memory improvement, and changes in neuronal survival are associated with changes in synaptic markers and mature dendritic spine density

To test if microglial repopulation might be linked to synaptic remodeling, we examined gene expression levels of pre- and post-synaptic markers upon the reappearance of microglia (Fig. 5f). There were no differences between the groups in the expression of pre- or post- synaptic markers (*Vglut1*, *Glua2*, *Glun2a*), markers of microglial-neuronal interaction (*Cx3cl1*, *Cx3cr1*), or neurotrophic factors (*Bdnf*, *Ngf*). We did see increased hippocampal expression of a microglial recruitment marker, *Cxcl10*, as well as elevated expression of *C1q*, the initiating protein of the canonical complement cascade, and *C3*; both of which are known to localize to synapses and mediate the elimination of dendritic spines by phagocytic microglia [35]. In accordance with this finding, the density of both synaptophysin and PSD-95 in the hilus was significantly reduced with microglial repopulation after ablation in the *Cx3cr1-Dtr* rats (Fig. 5g–j).

To examine whether microglial repopulation could affect hippocampal dendritic arborization or the density of spines on hippocampal pyramidal neurons, we stained hippocampal sections with FD Rapid GolgiStain. There was a significant increase in arborization of the basal dendrites in the CA3 in the *Cx3cr1*-*Dtr* rats compared to wt (Fig. 5n), but no differences in apical or basal dendritic tree length in this region (Fig. 5m) and no differences in the CA1 (Fig. 5k, l). Microglial repopulation also significantly increased the number of mature bifurcated spines, typically associated with increased spine efficacy [36, 37], in the CA1 in *Cx3cr1*-*Dtr* rats compared to wt (Fig. 5o, s). When the data were assessed as 18 to 28 dendritic segments per animal, we also revealed a significant reduction in stubby spines in the *Cx3cr1*-*Dtr* rats relative to wt (Fig. 5p). There were no differences in other spine types, and no differences in the CA3 (Fig. 5p).

### Repopulated microglia have a less complex morphology

**Fig. 6:**
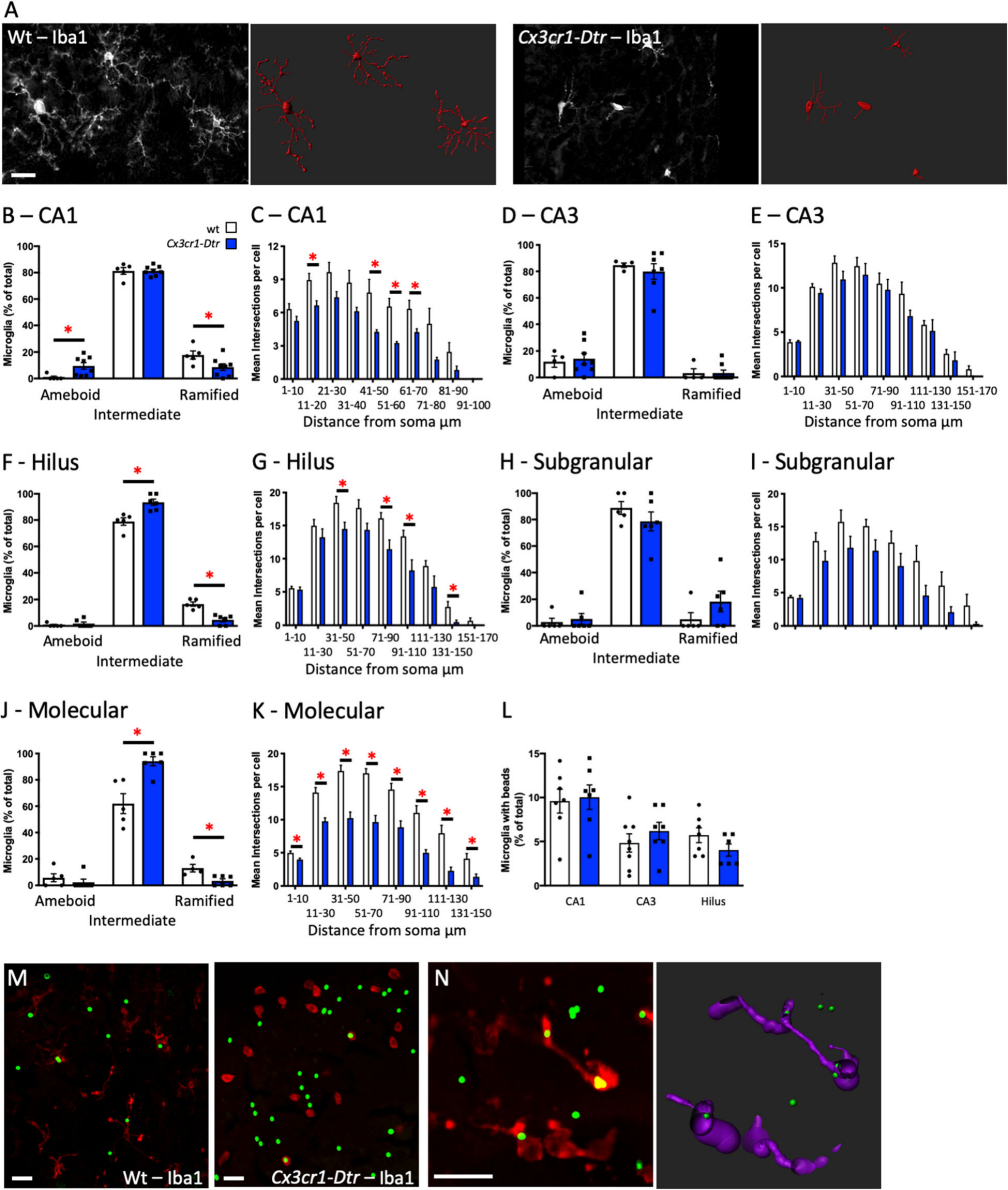
Repopulated microglia have a less complex morphology. Microglial profiles at 7 days after diphtheria toxin injection to ablate microglia in *Cx3cr1*-*Dtr* rats compared to wild-types (wt). **a** Representative inverted photomicrographs (white) and reconstructions (red) of microglial profiles generated in Imaris illustrating ramified and ameboid microglia. **b** Classification of repopulated microglia morphology in the CA1 (increased ameboid 0–1 projections; *U* = 3, *p =* 0.0085; *n* = 5–8). **c** Assessment of CA1 microglial complexity with Sholl analysis (distance from soma by genotype effect (*F*_(11,66)_ = 2.76, *p* = 0.005; *n* = 5–6). **d** CA3 classification. **e** CA3 Sholl analysis. **f** Hilus classification (increased intermediate 2–4 projections; *U* = 0, *p =* 0.0043; fewer ramified 5+ projections; *U* = 0, *p =* 0.0043). **g** Hilus Sholl analysis (distance from soma by genotype effect (*F*_(8, 56)_ = 4.44, *p* < 0.001; *n* = 5). **h** Subgranular/granular classification. **i** Subgranular/granular Sholl analysis. **j** Molecular classification (increased intermediate 2–4 projections; *U* = 3, *p =* 0.017; fewer ramified 5+ projections; *U* = 0, *p =* 0.0095). **k** Molecular Sholl analysis (distance from soma by genotype effect (*F*_(7, 49)_ = 10.17, *p* < 0.001; *n* = 5). **l** Microglial phagocytosis of polystyrene beads in the CA1 region. **m** Representative photomicrographs of Iba-1-positive cells with phagocytosed microbeads (block arrows). **n** Example of a microglial cell and its reconstruction showing co-localization of the beads inside the cell. Internalization was verified by imaging the region as a z-stack, transforming the z-stack into a 3D image and visually ensuring that the maximum fluorescence intensity started from within the cell. *p < 0.05; **b**, **d**, **f**, **h**, **j** Mann-Whitney *U* tests; **c**, **e**, **g**, **i**, **k** repeated measures ANOVA followed by Student’s unpaired *t* tests; **l** Student’s unpaired *t* tests. Data are expressed as mean ± SEM. Scale bars A = 50 μm; H = 20 μm; I = 10 μm

Since we saw differences in neuronal markers in association with improved memory function when the microglia had repopulated the brain, we next asked if we could identify differences in these repopulated microglia relative to controls. At 7 days after ablation, repopulated hippocampal microglia showed evidence of a less complex morphology (Fig. 6). In the CA1 region, there was an increase in the percentage of microglia classified as ameboid (Fig. 6a, b), as well as a reduction in complexity as assessed with Sholl analysis (Fig. 6c). In the hilus and molecular regions, we saw a similar pattern with more intermediate microglia and fewer ramified microglia (Fig. 6f, j), as well as reduced complexity (Fig. 6g, k) with no differences in the CA3 or subgranular/granular regions (Fig. 6d, e, h, i). We found similar changes in other memory-associated brain regions, including the dysgranular and granular retrosplenial cortex, perirhinal cortex, and the BLA, but not the CeA. In each case, except the CeA, there were no differences in microglial numbers between the wt and *Cx3cr1*-*Dtr* DT-treated rats at 7 days after ablation, but there was a shift toward a less complex morphology in these cells (Additional file 1: Table S1). It is likely that this morphology relates to the activation state of the microglia, since genes expressed by immature (embryonic and early postnatal) microglia were not elevated at this time (Additional file 4: Figure S3).

### Microglial repopulation does not increase their capacity for phagocytosis

Given that the repopulated microglia displayed an apparently “activated” morphology, we hypothesized that they might show differences in phagocytic activity that could account for the decrease in synaptophysin and PSD-95 that we see. However, phagocytosis of microbeads in a slice preparation was not different in repopulated hippocampal microglia from those in wt controls (Fig. 6l–n).

**Fig. 7:**
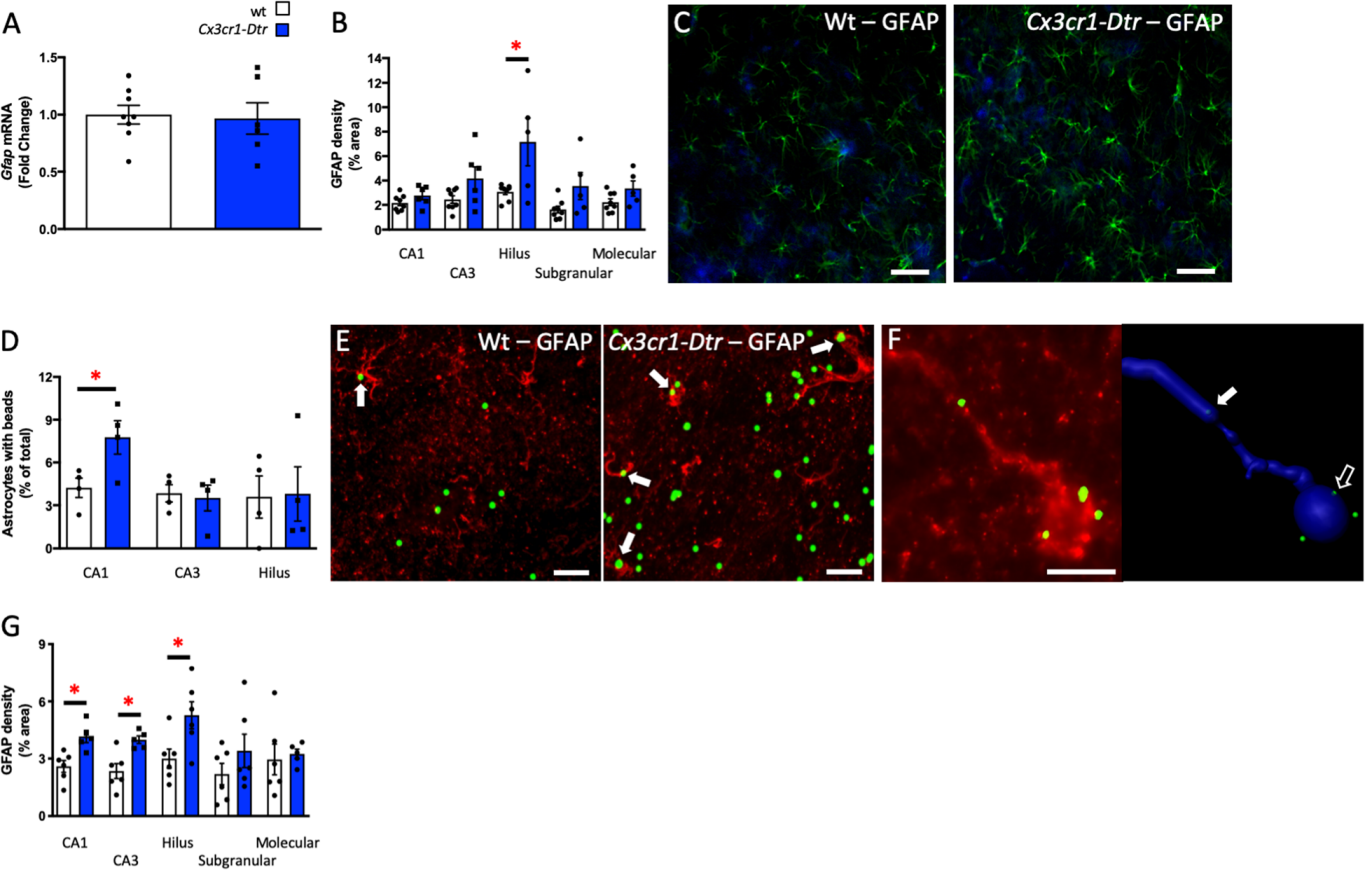
Microglial repopulation is associated with increased astrocyte activity. Astrocyte profiles at 7 days after diphtheria toxin injection to ablate microglia in *Cx3cr1*-*Dtr* rats compared to wild-types (wt). **a** Hippocampal expression of *Gfap*. **b** hippocampal astrocyte density (hilus: *t*_(11)_ = 2.67, *p =* 0.022. The *p* values in the CA3, subgranular/granular and molecular regions for the comparison between the wt and *Cx3cr1*-*Dtr* groups were 0.073, 0.060, and 0.088 respectively). **c** Representative photomicrographs of GFAP. **d** Astrocyte phagocytosis of polystyrene beads in the CA1, CA3, and Hilus regions (*t*_(6)_ = 2.61, *p =* 0.040). **e** Representative photomicrographs of GFAP-positive cells with phagocytosed microbeads (block arrows). **f** Example of an astrocyte and its reconstruction showing co-localization of the beads inside the cell (block arrow) and in the process of centering the cell (open arrow). Internalization was verified by imaging the region as a z-stack, transforming the z-stack into a 3D image and visually ensuring that the maximum fluorescence intensity started from within the cell. **g** Astrocyte profiles at 2 days after diphtheria toxin injection (CA1: *t*_(9)_ = 3.51, *p =* 0.007; CA3: *t*_(9)_ = 5.52, *p =* 0.007; hilus: *t*_(10)_ = 2.62, *p =* 0.025; *n* = 6 per group). *p < 0.05; Student’s unpaired *t* tests. Data are expressed as mean ± SEM. Scale bars C = 50 μm; E = 20 μm; F = 10 μm

Since generally elevated microglial phagocytosis upon repopulation did not seem to be responsible for the synaptic changes that we observed, we next assessed if microglial repopulation could influence astrocytes. We saw no differences in the expression of *Gfap* in the hippocampus (Fig. 7a). However, there was a significant increase in GFAP density in the hilus with microglial repopulation and a similar tendency in the CA3, subgranular/granular and molecular regions (Fig. 7b, c). Notably, hippocampal CA1 astrocytes in the brains of rats with repopulated microglia phagocytosed significantly more microbeads than those in wt controls (*t*_(6)_ = 2.61, *p =* 0.040; Fig. 7d–f), indicative that compensatory or reactive changes in astrocytes may be contributing to the neuronal and cognitive phenotype we see. In suggestion that the memory improvement at 7 days after microglial ablation is not exclusively an astrocyte effect, but is also related to the microglial repopulation itself, we also see increases in GFAP density throughout the hippocampus at day two, when no memory changes are present (Fig. 7g).

### Initial changes to neuronal number and memory dissipate as microglial and astrocyte morphology normalize

**Fig. 8:**
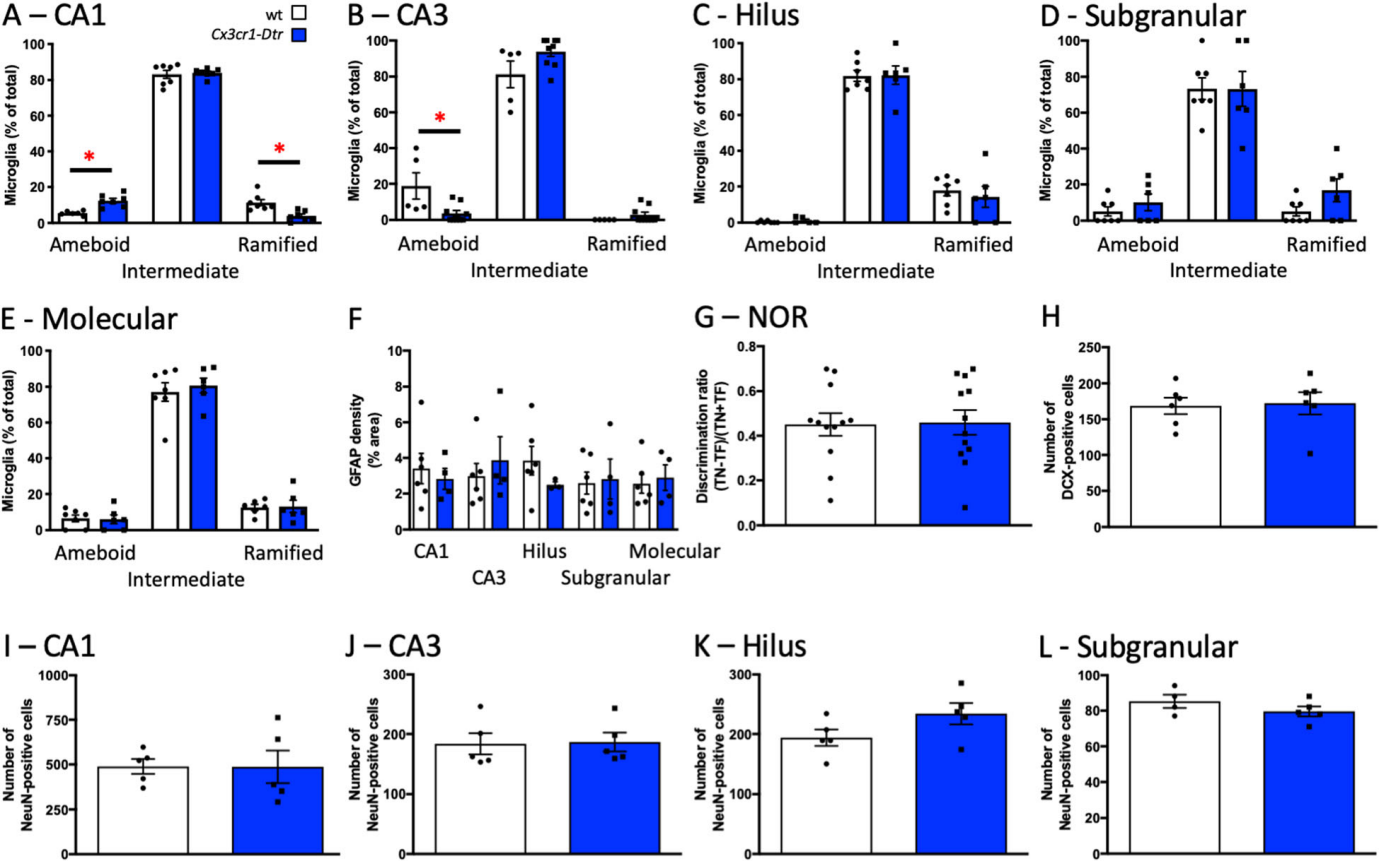
Initial changes to neuronal number and memory dissipate as microglial morphology normalizes. Microglial profiles, memory performance, and neuronal markers at 14 days after diphtheria toxin injection to ablate microglia in *Cx3cr1*-*Dtr* rats compared to wild-types (wt). **a** Classification of repopulated microglia morphology in the CA1 (increased ameboid; *U* = 0, *p =* 0.0012; fewer ramified; *U* = 1, *p =* 0.0012). **b** CA3 (fewer ameboid; *U* = 6, *p =* 0.024). **c** Hilus. **d** Subgranular/granular. **e** Molecular regions of the dentate gyrus. **f** Hippocampal astrocyte (glial fibrillary acid protein; GFAP) density. **g** Novel object recognition discrimination ratio (*n* = 11–12). **h** Number of DCX-positive cells in the subgranular/granular region of the dentate gyrus. **i** Number of NeuN-positive cells in the CA1. **j** CA3. **k** Hilus. **l** Subgranular/granular region of the dentate gyrus (*n* = 5–6). *p < 0.05; **a**–**e**: Mann-Whitney *U* tests. Data are expressed as mean ± SEM

To determine if the enhanced memory performance and associated neuronal changes with microglial repopulation are sustained long-term, we examined short-term memory and neuronal markers 14 days after DT, at which point the microglia had fully repopulated the brain. At this time, there continued to be more microglia in the CA1 with an ameboid morphology and fewer that were ramified (Fig. 8a), but there were fewer ameboid microglia in the CA3 (Fig. 8b) and no differences in the other regions of the hippocampus (Fig. 8c–e). At this time, astrocyte density had also recovered to wt levels (Fig. 8f). In the NOR task, there were no differences in performance in the acquisition (Additional file 3: Figure S2) or test phase (Fig. 8g) at this time point. There were also no differences in the number of immature or mature neurons in the hippocampus (Fig. 8h–l), suggesting any effect of microglial remodeling on memory performance is transient and likely related to the microglial repopulation or effects on astrocytes.

### Short-term memory enhancement and neuronal correlates are specifically related to the microglial repopulation phase

**Fig. 9:**
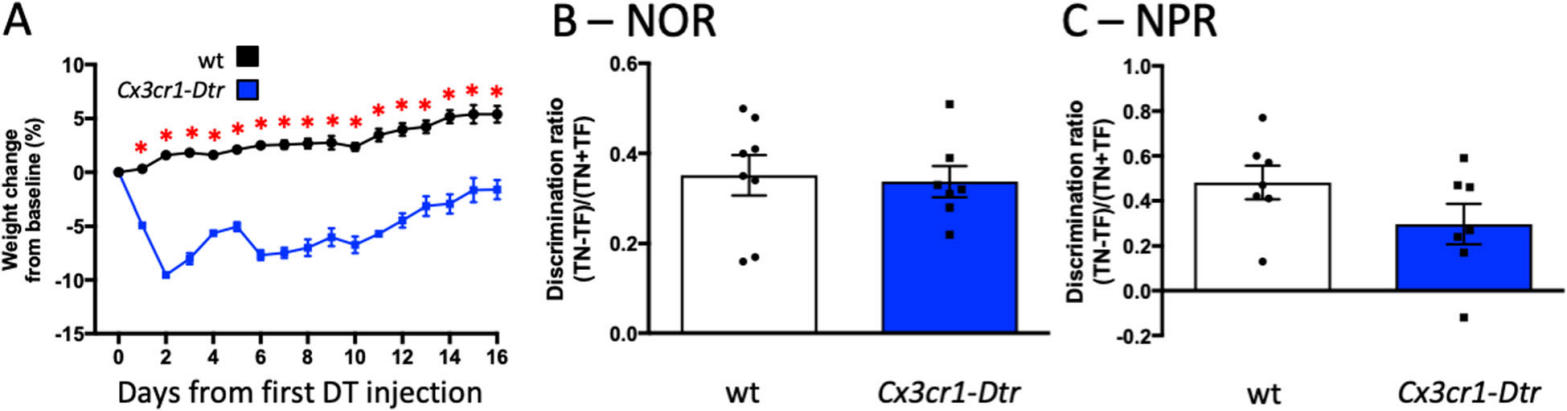
Short-term memory enhancement is specifically related to microglial repopulation. Memory performance at 48 h after chronic microglial depletion with diphtheria toxin injection in *Cx3cr1*-*Dtr* rats compared to wild-types (wt). **a** Weight change with DT injection (*F*_(15,210)_ = 6.16, *p* = 0.026). **b** Novel object recognition discrimination ratio in wild-type (wt) and *Cx3cr1*-*Dtr* rats (*n* = 7–8). **c** Novel place recognition discrimination ratio (*n* = 7 per group). *p < 0.05; repeated measures one-way ANOVA followed by Student’s unpaired *t* tests. Data are expressed as mean ± SEM

To determine whether the memory improvement seen at 7 days post-DT in our *Cx3cr1*-*Dtr* rats is due to a prolonged effect of the microglial ablation, or, rather, to the microglial repopulation and its associated effects, we chronically ablated microglia and assessed the rats’ performance in the NOR and NPR tasks 48 h after the last injection (Fig. 9). Body weight remained reduced with multiple exposures to DT, indicative that the microglia were indeed chronically ablated [22], although we did not examine microglial numbers in this cohort. Consistent with acute microglial ablation, the *Cx3cr1*-*Dtr* rats showed no significant differences in the duration spent exploring the novel object (positive discrimination ratio) from the wt rats in the NOR and no differences in the NPR at 16 days after microglial ablation if the microglia remained depleted. These data suggest that there is a short-term memory improvement specifically associated with microglial repopulation after depletion.

## Discussion

Microglia are crucial in programming normal development of the brain [13–16, 38] and fundamental in responding to pathogens and injury in adulthood [13, 39–41]. Yet, remarkably, memory is not adversely affected in the absence or suppression of these cells. Our data show that with acute microglial ablation, when the population was reduced to less than 17% of controls throughout the hippocampus, memory in the Y maze and NOR tasks was normal. Similarly, when the microglia remained chronically ablated for more than 2 weeks, NOR and NPR performance was normal. Other groups have shown similar findings in other species with suppression of microglia with colony-stimulating factor 1 receptor (CSF1R) inhibitors causing no deficits in learning and memory [42–45].

Not only was there no apparent detrimental effect of microglial ablation on short-term memory, there was even a beneficial, if transient, outcome for memory once the microglia had repopulated the brain. Thus, at 7 days after initial ablation, at which time microglial numbers had returned to normal but morphology indicated that these cells may be more highly activated, memory in the NOR and NPR tasks was improved. Performance in these memory tasks is unlikely to be related to novelty preference, since both groups of rats explored the objects to a similar degree in the acquisition task. They also both displayed similar degrees of locomotor and exploratory behavior in the Y maze, NOR, and NPR tasks. Improved cognitive performance after microglial repopulation has previously been reported in aged mice in a CSFR1-inhibition model [45], but this group saw no cognitive benefit in young adults, suggesting microglial genes that remain active with PLX5622, species differences, or test conditions may be important in conferring or revealing the memory effects of microglial repopulation. It is worth noting that in addition to depleting microglia, our model also results in ablation of circulating (but not spleen) monocytes, with a similar time course of depletion and repopulation as that seen in microglia [22]. While it seems unlikely monocyte depletion is principally responsible for the central effects we see, this caveat should be considered in the interpretation of our data. We should also consider when interpreting data from our model that we have not yet established how microglial debris is being cleared after DT treatment or how microglia repopulate the brain. Studies in mice with PLX compounds which target CSFR1 suggest that microglia die by apoptosis without an inflammatory component [46] and our own data suggest there is no peripheral or central inflammation at the peak of depletion or during repopulation [22]. Since we see an increase in GFAP density during this repopulation, it is likely astrocytes play a role in restoring the microglia but this remains to be fully investigated.

Our data showing a transiently improved memory with microglial repopulation also establish that there are corresponding differences in synaptic pruning and neuronal remodeling at the same time. It is well established that microglia participate in adaptive synaptic pruning during development [14–16]. It has more recently become apparent that microglia are also responsible for the detrimental synaptic pruning that occurs during obesity in adults and leads to cognitive dysfunction [8]. In this case, microglia may hyper-phagocytose synaptic spines and preventing this phagocytosis normalizes the poor cognitive function [8]. The dendritic synaptic spine is responsible for integrating synaptic inputs; it is thought that mature spines become strengthened when the dendrite is stimulated, providing the physical substrate for effective propagation of signals and ultimately memory storage. An increased density of synaptic spines is thus associated, in most cases, with better memory [47]. Yet, females show fluctuations in spine density by up to 30% across the ovarian cycle but can display equal success in memory tasks while employing different recall strategies [48, 49]. Thus, it is probable that the balance between mature and immature spines is a key encoder for successful memories. Our data from the current study suggest that neuronal remodeling is impacted as microglia repopulate the brain after ablation, but that the relationship is not simple. Our measures of synaptophysin and PSD-95 are in accordance with our phagocytosis assay of astrocytes, suggesting hyper-phagocytosis of synaptic elements. In contrast, we see more mature neurons, an increased dendritic tree branching, and more bifurcated dendritic spines, suggesting phagocytosis might be reduced. It is likely that both are occurring but that the specific signals for which elements are phagocytosed are disrupted. Potentially, microglia are capable of phagocytosing (such as with the microbeads) but are unable to respond to the neuronally expressed complement on mature (bifurcated) [50, 51] spines, which would account for why complement genes are over-expressed. Astrocytes may also be hyper-phagocytic in an indiscriminate manner, which could account for the reduction in PSD-95 and synaptophysin density, but not be reflected in the spine counts since intact segments of dendrites were assessed in each case. We suggest that such strategic and localized circuit refinement is important for the improved short-term memory function that we see.

A key finding of the current study is the astrocyte response to microglial repopulation, leading to the suggestion that astrocytes in combination with microglia may be responsible for improved memory. Here, we showed that microglial repopulation led to an increased density of astrocytes throughout the hippocampus. Astrocyte density had fully normalized at the 14-day time point (when memory was also normal). Most importantly, while the phagocytic activity of the repopulated hippocampal microglia was not different from that of wt animals, astrocytes in the same region phagocytosed twice as many microbeads. Astrocytes also communicate with neurons and have recently been shown to regulate synaptic formation, transmission, and plasticity; enhancing learning and memory [52–57]. For instance, astrocyte activation in the CA1 can enhance learning-specific neuronal activation and memory recall [56]. In doing this, they make contact with synapses in a neuronal activity-dependent manner [57]. They actively engulf synapses, curtailing growth in those they contact [53], and mediating their elimination in a MEGF10 and MERTK-dependent phagocytic mechanism [57]. As such, conditional ablation of astrocytes leads to a reduction in cortical gamma oscillations as well as specific impairments in the NOR [52]. These data suggest that any neuronal circuit remodeling in our model may be due in part to the astrocyte response to the microglial repopulation. Notably, astrocyte density was also increased at 2 days after microglial ablation, when no memory enhancement was seen, suggesting a combined microglial and astrocyte effect is needed to induce changes in memory. We also note that our phagocytosis assay assesses capacity to take up synthetic microbeads, which do not express complement or other specific targets for engulfment. Thus, these data do not rule out that microglia may hyper- or hypo-phagocytose dendritic spines in a complement-dependent manner in vivo in this model. This, and the specific involvement of astrocyte-dependent synaptic pruning remain to be further investigated in future work.

It is important to note that microglial and astrocyte activation in other states (inflammation, high-fat diet, Alzheimer’s disease, stroke) is not usually thought to be beneficial to memory. Indeed, we have seen that increased microglial density as a result of neonatal overfeeding is associated with poor memory [21]. However, in many of these cases, it is difficult to tease the cause from the effect. In some instances, the negative impacts on cognition are due to a primary cause of injury (e.g., stroke causing neuronal apoptosis) and glia are important in mitigating this effect [58]. In other cases, such as with obesity and aging, glial activation becomes prolonged potentially leading to indiscriminate synaptic pruning [8, 59, 60]. It is an important consideration that our model of microglial ablation is not associated with an inflammatory response such as is seen with high-fat diet, stroke, etc. We see no peripheral or central increases in cytokine gene expression or cytokine levels, and no evidence of sickness behavior at any assessed time after microglial ablation [22]. The differences between the short-memory effects seen in our study and in cases of microglial and astrocyte activation with an inflammatory component, like stroke and high-fat diet, are likely related to this inflammation.

It is also noteworthy that our investigation was restricted to the effects of microglial ablation on short-term memory during the daytime. Future studies examining performance in a probe trial for the NOR and NPR would be informative as to whether microglia are necessary for memory consolidation as will tasks that require memory consolidation over the long-term. Indeed, recent study has shown that microglia show diurnal variation in morphology, even when unstimulated, with increased branching/complexity in the active phase (night in rats) relative to the inactive [61]. Notably, cortical microglia exhibit a circadian expression of cathepsin S (CatS), a microglia-specific lysosomal cysteine protease. This protease is secreted during the active phase to decrease the spine density of cortical neurons leading to a reduction in synaptic strength during the following inactive phase [62] and a selective elimination of synaptic proteins during sleep [63]. Thus, tasks that incorporate one or more sleep phases may reveal that the absence of microglia compromises memory and this remains to be explored.

## Conclusions

Our model provides a useful tool to study the role of adult microglia (without the complicating addition of developmental microglial ablation) with an acute ablation (without the need for protracted administration of pharmacological agents). Our data add to the understanding of the role of these immune cells in memory, highlighting a novel contribution in the healthy adult. Although microglial repopulation may recruit other memory-modulating mechanisms and involve brain regions other than hippocampus, our data here suggest that acute non-inflammatory astrocyte activation in healthy individuals, that occurs as microglia repopulate the brain after ablation, leads to transiently improved short-term memory. These data suggest that microglial and astrocyte dynamics may play a role in selectively enhancing memory. These cell types may therefore present useful targets for treatment strategies to enhance short-term memory. PLX compounds allowing renewal or rejuvenation of the brain’s microglial population have proven in mice to be beneficial for cognition in ageing [45] and may be a useful strategy in humans. However, the long-term implications for microglial ablation on the brain’s capacity to respond to pathogens and injury remain to be determined.

## Supplementary information

**Additional file 1:Table S1.** Microglial numbers and morphology in memory-associated brain regions at 7 days after ablation.

**Additional file 2: Figure S1.***Dtr* expression, in the absence of DT, did not affect any measured parameters. Expression of *Dtr* did not affect the number of microglia through the A) CA1, B) CA3, C) hilus, D) subgranular / granular, or E) molecular regions of the dentate gyrus, in *Cx3cr1-Dtr* rats relative to wild-types (wt; *n* = 5–6 per group). It did also not affect F) hippocampal *Cx3cr1* expression (*n* = 6 per group), G) Y maze latency to arm entry, H) Y maze total arm entries, I) Y maze discrimination ratio, J) the novel object recognition discrimination ratio. In the Y maze, both groups of rats had a positive discrimination ratio that was significantly different from zero in one-sample t-tests and there were no group differences between the wt and *Cx3cr1-Dtr* groups (one sample t-test to zero: wt: t _(6)_ = 3.08, *p* = 0.022. *Cx3cr1-Dtr:* t _(6)_ = 2.99, *p* = 0.025). In the novel object recognition task, both groups had a positive discrimination ratio that was significantly different from zero (wt: t _(6)_ = 8.79, *p* = 0.0001. *Cx3cr1-Dtr*: t _(6)_ = 4.39, *p* = 0.0046) with no differences between the groups (n = 6–7 per group). * *p* < 0.05: Student’s unpaired t-tests. Data are expressed as mean ± SEM.

**Additional file 3:Figure S2.** Microglial ablation and repopulation did not affect overall locomotor activity or exploration in the Y maze, novel object recognition (NOR) or novel place recognition (NPR) tasks. Microglial ablation did not affect locomotor activity or exploration at A, B) 48 h, C-E) 7 days, or F, G) 14 days in the Y maze, NOR or NPR. No significant differences with Student’s unpaired t-tests. Data are expressed as mean ± SEM.

**Additional file 4: Figure S3.** Microglial repopulation is not associated with increased expression of immature microglial markers. Microglial ablation did not affect embryonic or foetal/early postnatal microglial genes. Genes expressed in adult microglia, *Cd14* (t _(9)_ = 2.70, *p =* 0.024) and *Mef2a* (t _(9)_ = 2.39, *p =* 0.041) were increased at 7 days after microglial ablation. * *p* < 0.05: Student’s unpaired t-tests. Data are expressed as mean ± SEM.

## Data Availability

The datasets used and/or analyzed during the current study are available from the corresponding author on reasonable request.

## Abbreviations

BLA: Basolateral amygdala
CeA: Central amygdala
DCX: Doublecortin
DT: Diphtheria toxin
Dtr: Diphtheria toxin receptor
GFAP: Glial fibrillary acidic protein
i.p.: Intraperitoneal
Iba-1: Ionized calcium binding adaptor molecule 1
ITI: Inter-trial interval
NeuN: Neuronal nuclei
NOR: Novel object recognition
NPR: Novel place recognition
PSD: Post-synaptic density
RsC: Retrosplenial cortex
s.c.: Subcutaneous
Wt: Wildtype
