# Supplementary material for: Glial remodeling enhances short-term memory performance in Wistar rats
Source: J Neuroinflammation. 2020 Feb 7;17:52. doi: 10.1186/s12974-020-1729-4 (PMC7006153; doi:10.1186/s12974-020-1729-4)
Supplement: Supplementary file 1 — Additional file 1:Table S1. Microglial numbers and morphology in memory-associated brain regions at 7 days after ablation. [file 12974_2020_1729_MOESM1_ESM.docx]

**Supplementary Table 1.** Microglial numbers and morphology in memory-associated brain regions at 7 days after ablation.

|  | **Wt** | ***Cx3cr1-Dtr*** | **Statistical analysis** |
| --- | --- | --- | --- |
| **Dysgranular Retrosplenial Cortex** | |  |  |
| *Number* | 67.5 ± 4.6 | 76.3 ± 5.1 | NSD |
| *Ameboid (%)* | 28.1 ± 3.2 | 30.0 ± 1.7 | NSD |
| *Intermediate (%)* | 41.9 ± 1.6 | 54.8 ± 3.7 * | t_(10)_ = 3.23; *p* = 0.009 |
| *Ramified (%)* | 30.0 ± 3.8 | 15.2 ± 2.4 * | t_(10)_ = 3.27; *p* = 0.0084 |
| **Granular Retrosplenial Cortex** | |  |  |
| *Number* | 74.2 ± 7.0 | 69.5 ± 3.0 | NSD |
| *Ameboid (%)* | 15.0 ± 1.8 | 22.2 ± 2.0 * | t_(10)_ = 2.66; *p* = 0.024 |
| *Intermediate (%)* | 44.7 ± 3.5 | 60.4 ± 1.9 * | t_(10)_ = 3.92; *p* = 0.0029 |
| *Ramified (%)* | 40.3 ± 4.2 | 17.4 ± 2.1 * | t_(10)_ = 4.90; *p* = 0.0006 |
| **Perirhinal Cortex** | |  |  |
| *Number* | 161.9 ± 3.1 | 175.3 ± 7.2 | NSD |
| *Ameboid (%)* | 24.4 ± 2.9 | 33.3 ± 4.2 | NSD |
| *Intermediate (%)* | 49.5 ± 1.5 | 69.5 ± 2.2 * | t_(10)_ = 7.67; *p* = 0.0001 |
| *Ramified (%)* | 26.1 ± 3.5 | 24.7 ± 3.3 | NSD |
| **Basolateral Amygdala** | |  |  |
| *Number* | 65.3 ± 5.0 | 88.7 ± 5.3 | NSD |
| *Ameboid (%)* | 31.1 ± 2.0 | 30.4 ± 4.3 | NSD |
| *Intermediate (%)* | 42.2 ± 2.4 | 50.5 ± 2.4 * | t_(9)_ = 2.46; *p* = 0.036 |
| *Ramified (%)* | 25.9 ± 1.7 | 19.1 ± 2.0 * | t_(9)_ = 2.44; *p* = 0.037 |
| **Central Amygdala** |  |  |  |
| *Number* | 33.1 ± 5.0 | 26.1 ± 4.3 | NSD |
| *Ameboid (%)* | 41.3 ± 2.6 | 44.3 ± 2.2 | NSD |
| *Intermediate (%)* | 25.4 ± 4.1 | 29.7 ± 3.5 | NSD |
| *Ramified (%)* | 33.1 ± 5.0 | 26.1 ± 4.3 | NSD |
