# Supplementary figures and images for: Glial remodeling enhances short-term memory performance in Wistar rats
Source: J Neuroinflammation. 2020 Feb 7;17:52. doi: 10.1186/s12974-020-1729-4 (PMC7006153; doi:10.1186/s12974-020-1729-4)

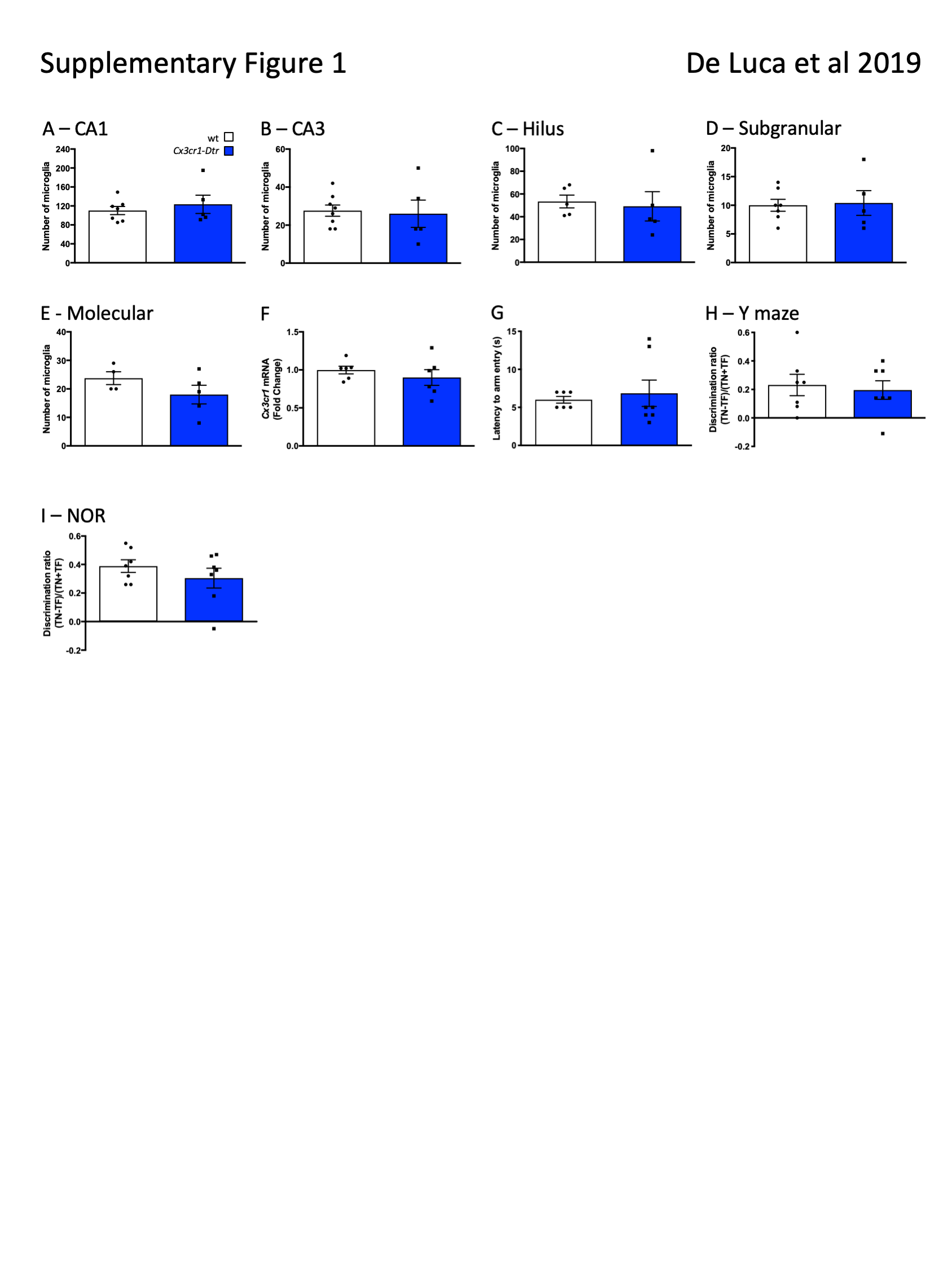

Supplement: Supplementary file 2 — Additional file 2: Figure S1.Dtr expression, in the absence of DT, did not affect any measured parameters. Expression of Dtr did not affect the number of microglia through the A) CA1, B) CA3, C) hilus, D) subgranular / granular, or E) molecular regions of the dentate gyrus, in Cx3cr1-Dtr rats relative to wild-types (wt; n = 5–6 per group). It did also not affect F) hippocampal Cx3cr1 expression (n = 6 per group), G) Y maze latency to arm entry, H) Y maze total arm entries, I) Y maze discrimination ratio, J) the novel object recognition discrimination ratio. In the Y maze, both groups of rats had a positive discrimination ratio that was significantly different from zero in one-sample t-tests and there were no group differences between the wt and Cx3cr1-Dtr groups (one sample t-test to zero: wt: t (6) = 3.08, p = 0.022. Cx3cr1-Dtr: t (6) = 2.99, p = 0.025). In the novel object recognition task, both groups had a positive discrimination ratio that was significantly different from zero (wt: t (6) = 8.79, p = 0.0001. Cx3cr1-Dtr: t (6) = 4.39, p = 0.0046) with no differences between the groups (n = 6–7 per group). * p < 0.05: Student’s unpaired t-tests. Data are expressed as mean ± SEM. [file 12974_2020_1729_MOESM2_ESM.tiff]

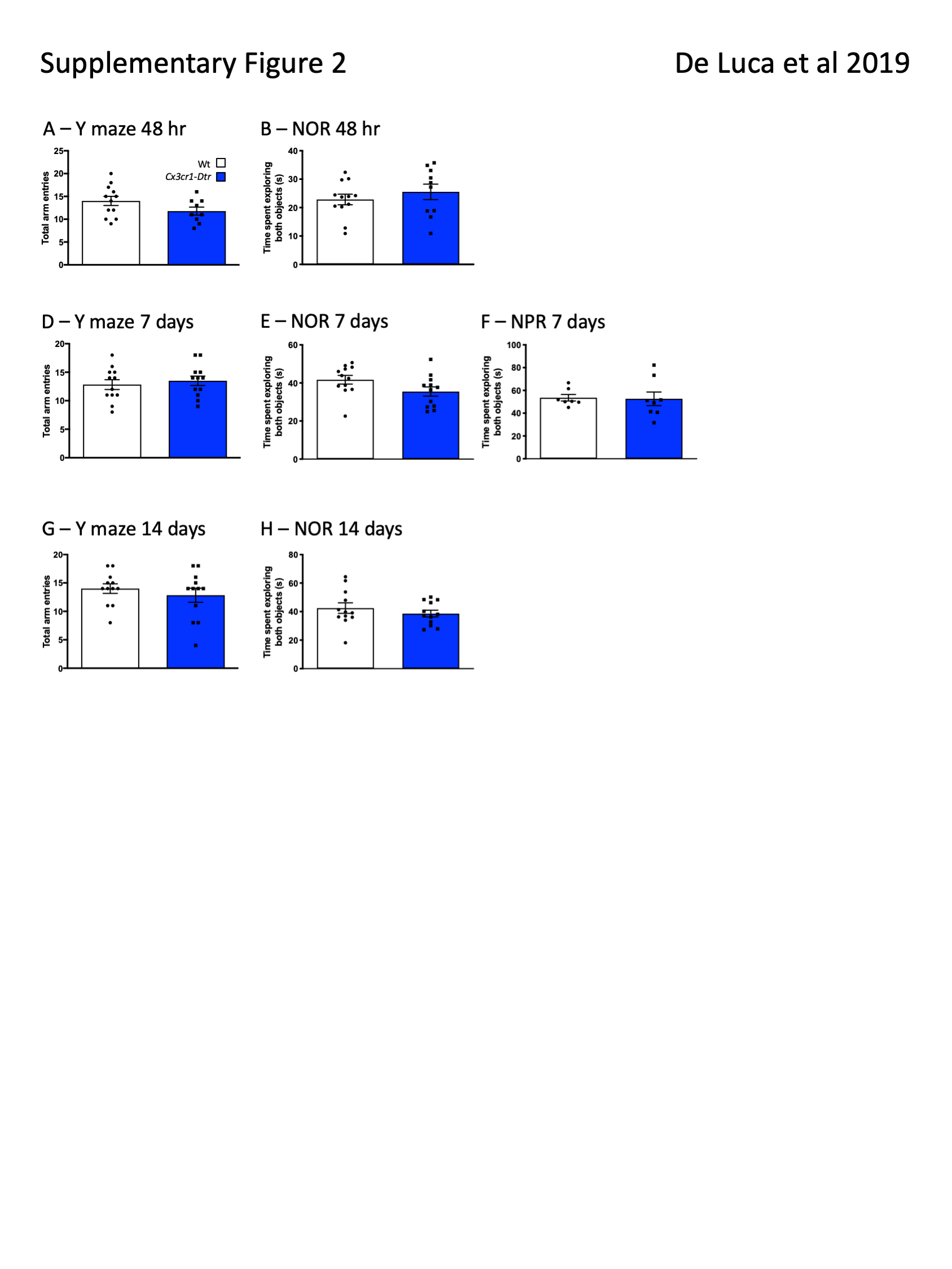

Supplement: Supplementary file 3 — Additional file 3:Figure S2. Microglial ablation and repopulation did not affect overall locomotor activity or exploration in the Y maze, novel object recognition (NOR) or novel place recognition (NPR) tasks. Microglial ablation did not affect locomotor activity or exploration at A, B) 48 h, C-E) 7 days, or F, G) 14 days in the Y maze, NOR or NPR. No significant differences with Student’s unpaired t-tests. Data are expressed as mean ± SEM. [file 12974_2020_1729_MOESM3_ESM.tiff]

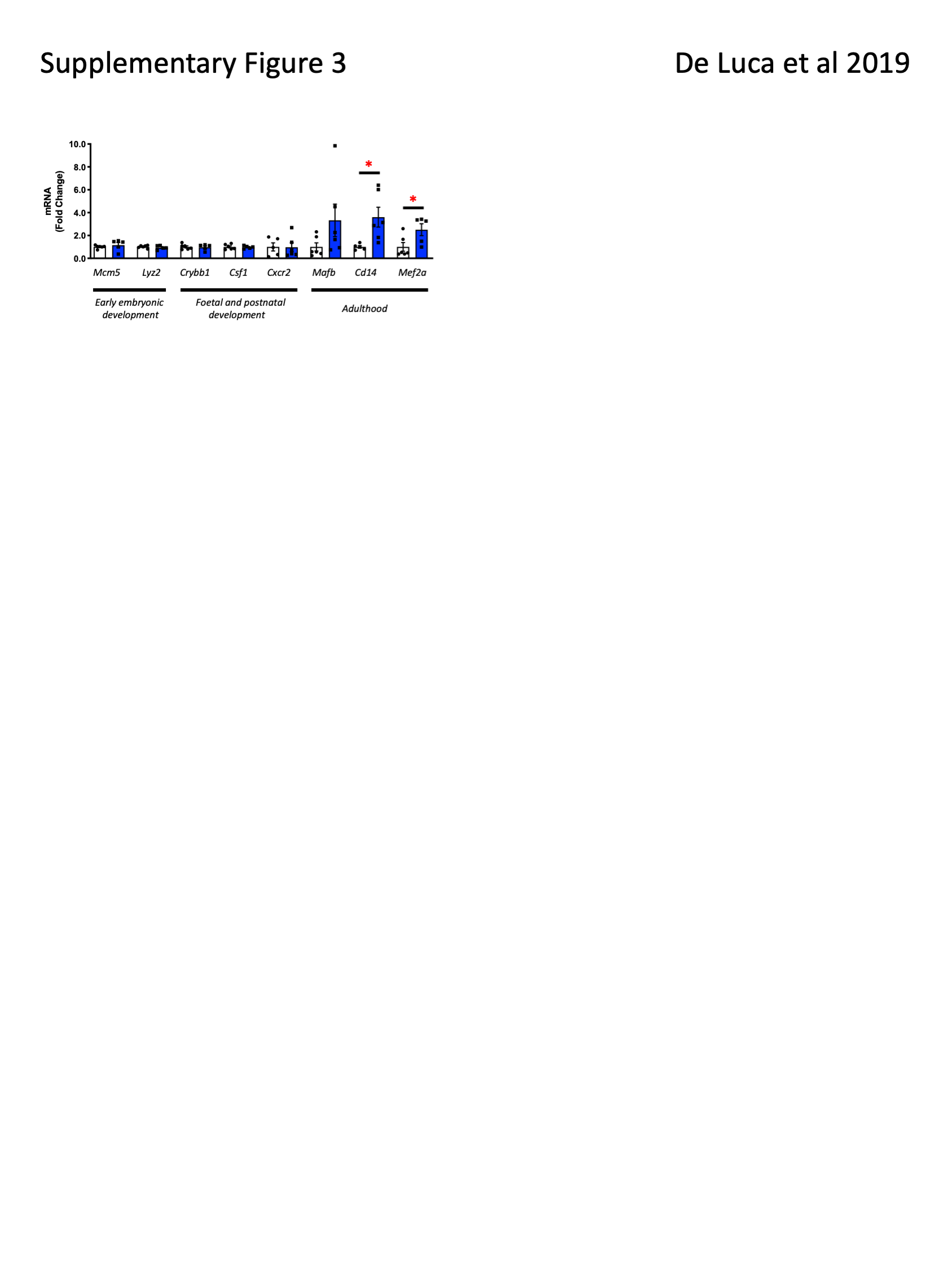

Supplement: Supplementary file 4 — Additional file 4: Figure S3. Microglial repopulation is not associated with increased expression of immature microglial markers. Microglial ablation did not affect embryonic or foetal/early postnatal microglial genes. Genes expressed in adult microglia, Cd14 (t (9) = 2.70, p = 0.024) and Mef2a (t (9) = 2.39, p = 0.041) were increased at 7 days after microglial ablation. * p < 0.05: Student’s unpaired t-tests. Data are expressed as mean ± SEM. [file 12974_2020_1729_MOESM4_ESM.tiff]
